# Effect of a single intra-articular injection of bupivacaine on synovial fluid prostaglandin E_2_ concentrations in normal canine stifles

**DOI:** 10.1186/s13104-018-3360-3

**Published:** 2018-04-25

**Authors:** Jenna E. Giangarra, Sabrina L. Barry, Linda A. Dahlgren, Otto I. Lanz, Marian E. Benitez, Stephen R. Werre

**Affiliations:** 1 Department of Small Animal Clinical Sciences, Virginia-Maryland College of Veterinary Medicine, Virginia Tech, 205 Duck Pond Drive, Blacksburg, VA 24061 USA; 2 Department of Large Animal Clinical Sciences, Virginia-Maryland College of Veterinary Medicine, Virginia Tech, 205 Duck Pond Drive, Blacksburg, VA 24061 USA; 3 Laboratory for Study Design and Statistical Analysis, Virginia-Maryland College of Veterinary Medicine, Virginia Tech, 205 Duck Pond Drive, Blacksburg, VA 24061 USA

**Keywords:** Bupivacaine, Stifle, Canine, Arthrocentesis, Synovitis, Prostaglandin E_2_

## Abstract

**Objective:**

To identify if synovial fluid prostaglandin E_2_ increases in response to a single intra-articular dose of bupivacaine in the normal canine stifle.

**Results:**

There were no significant differences in synovial fluid prostaglandin E_2_ (PGE_2_) concentrations between treatment groups or over time within bupivacaine or saline groups. Samples requiring ≥ 3 arthrocentesis attempts had significantly higher PGE_2_ concentrations compared to samples requiring 1 or 2 attempts. Following correction for number of arthrocentesis attempts, PGE_2_ concentrations were significantly higher than baseline at 24 and 48 h in the bupivacaine group; however there were no significant differences between the bupivacaine and saline groups. In normal dogs, a single bupivacaine injection did not cause significant synovial inflammation, as measured by PGE_2_ concentrations, compared to saline controls. Future research should minimize aspiration attempts and include evaluation of the synovial response to bupivacaine in clinical cases with joint disease.

## Introduction

Bupivacaine is a local anesthetic that works directly on the neuronal cell membrane, blocking impulse conduction in nerve fibers. When administered intra-articularly, as part of a multimodal analgesic protocol, bupivacaine provides long-lasting local pain relief after joint surgery in people and animals.

The safety of intra-articular bupivacaine came into question when clinical case reports concluded an association between intra-articular bupivacaine and glenohumeral chondrolysis, a rare complication following shoulder arthroscopy in people [1–3]. Subsequently, ex vivo studies have reported that bupivacaine causes chondrocyte death and that this chondrotoxicity is time and dose dependent [4]. Based on the results of these studies, researchers often conclude or assume that intra-articular bupivacaine causes clinical glenohumeral chondrolysis through this mechanism of direct chondrotoxicity.

The delay of several months between bupivacaine infusion and clinically evident chondrolysis suggests that chondrolysis may be a sequela to an extra-cartilaginous process (such as synovitis) that indirectly leads to chondrocyte toxicity or makes chondrocytes more vulnerable to subsequent insults. The idea that bupivacaine may cause acute synovitis is supported by studies that document signs of synovitis after local anesthetic injection in multiple species (lidocaine in bovine carpal joints, mepivacaine in equine carpal joints, bupivacaine in rabbit knee joints). Sustained synovitis can indirectly lead to cartilage degradation through the actions of enzymes, inflammatory mediators, and cytokines [5]. An insult to the joint induces release of pro-inflammatory cytokines from synoviocytes which in turn increase the synthesis of other inflammatory mediators from the synovium, such as prostaglandin E_2_ (PGE_2_). PGE_2_ is a marker of synovitis and stimulates expression of matrix metalloproteinases that play a major role in articular cartilage degeneration.

The objective of this study was to identify if there is a synovial response to a single intra-articular dose of bupivacaine in the normal canine stifle. We hypothesized that a single intra-articular bupivacaine injection would result in a detectable increase in synovial fluid PGE_2_ concentration compared to intra-articular saline injection.

## Main text

### Methods

Eight healthy adult male intact purpose bred Beagle dogs were purchased from Covance Inc. (Cumberland, VA) and housed in our institution in a manner approved by the institutional animal care and use committee at Virginia Tech. The mean age of the dogs was 1.59 years (range 1.57–1.62) and the mean weight was 12.1 kg (range 10.1–13.4). Dogs were included if they were deemed healthy based on normal physical and orthopedic examinations. Dogs were heavily sedated with intravenous (IV) hydromorphone (0.05 mg/kg) and dexmedetomidine (5 mcg/kg). Bilateral orthogonal stifle radiographs were obtained and a radiologist reviewed all radiographic images to ensure there was no evidence of stifle osteoarthritis.

Stifles were randomized using a random number generator^1^ such that each dog had one stifle allocated to bupivacaine and the contralateral stifle allocated to saline control treatments. Prior to sampling, dogs received a subcutaneous injection of a long acting cephalosporin, cefovecin sodium (8 mg/kg), to protect against bacterial contamination as a result of repeated arthrocentesis during this study. Synovial fluid was sampled at 5 time points: immediately prior to intra-articular injection (T0), 30 min following injection (T30), 60 min following injection (T60), 24 h following injection (T24) and 48 h following injection (T48).

The first 3 time points (T0, T30 and T60) were collected on the 1st day (Day 0). When necessary, dogs were administered additional sedation (dexmedetomidine 5 mcg/kg IV). A standard medial or lateral parapatellar arthrocentesis technique was used, based on dog positioning [6]. After acquiring the first sample (T0), a new syringe and needle were used to administer either 0.5% preservative-free bupivacaine in saline^2^ (0.2 mL/kg) or an equal volume of 0.9% saline. Following intra-articular injection, a digital timer was set for 30 min and 10 passive range of motion cycles were performed. Following the last sample collection, dogs were reversed with intramuscular atipamezole (equal volume to dexmedetomidine). The remaining time points (T24, T48) were collected on Days 1 and 2, respectively, using the same techniques. Following collection, all synovial fluid samples were transferred into microfuge tubes and stored at − 80 °C until batch analysis.

Within 6 months of collection, samples were thawed on ice and PGE_2_ quantified by competitive ELISA^3^ and read by spectrophotometer^4^ in accordance with the manufacturer’s instructions. Samples were diluted 1:2 with ELISA buffer and when necessary additional dilutions were performed to ensure results were within the standard curve of the plate.

#### Statistical analysis

Prior to the study, a power analysis was performed using data from an unpublished pilot study that measured the increase in PGE_2_ concentration 30 min following intra-articular injection of bupivacaine. A sample size of 6 stifles per group was estimated to achieve 83% power to detect a mean of paired differences of 480 pg/mL between groups with a significance level (alpha) of 0.05.

Data were analyzed using commercial software.^5^ T0 concentrations were corrected to account for the inherent dilution created by infusion of either bupivacaine or saline into the joint using the following formula (T0c):

$$T0c = \frac{T0 \times Vi}{Vf}.$$

where initial volume (Vi) equals 0.08 mL/kg [7] and final volume (Vf) equals (Vi − Vaspirated) + Vtreatment.

Normal probability plots showed that PGE_2_ concentrations were skewed and data are therefore summarized as median (range). A logarithmic transformation (base e) was applied to the concentrations before downstream data analysis. Effects of treatment and time were assessed using mixed-model repeated-measures ANOVA followed by Tukey’s procedure for multiple comparisons. Residuals were inspected to verify that the errors followed a normal distribution with constant variance. A post hoc analysis (mixed-model ANOVA) was performed to investigate the association between number of aspiration attempts required to collect an adequate sample and PGE_2_ concentration. This analysis revealed that ≥ 3 attempts resulted in significantly elevated PGE_2_ concentrations, and so a sensitivity re-assessment of the effects of treatment and time using data obtained after only 1 or 2 aspiration attempts was performed. Statistical significance was set at p < 0.05.

### Results

No radiographic evidence of osteoarthritis was identified in any stifle in any dog. There were no significant differences in PGE_2_ concentrations between the bupivacaine and saline groups (p = 0.229–0.898) or over time within each group (p = 0.152 and 0.343 for bupivacaine group and saline group, respectively) when all data were included in the analysis without consideration for number of aspiration attempts (Table 1).

**Table 1:** PGE_2_ concentrations in canine stifle synovial fluid collected following bupivacaine or saline injection

Time	Treatment	Sample size	Median (pg/mL)	Range
T0	Bupivacaine	8	287.1	58.3–734.9
T0	Saline	8	208.3	96.2–954.9
T30	Bupivacaine	8	244.2	128.9–2026.0
T30	Saline	8	269.9	91.7–430.1
T60	Bupivacaine	8	512.3	142.3–3669.5
T60	Saline	8	468.3	172.3–1074.2
T24	Bupivacaine	8	726.2	197.8–1675.8
T24	Saline	8	446.4	159.8–910.3
T48	Bupivacaine	8	391.6	210.0–1722.4
T48	Saline	8	390.2	118.5–5032.2

Data presented are without correction for number of arthrocentesis attempts

The number of aspiration attempts required to collect an adequate sample was 1 in the majority of cases (62/80), but was as high as 6 attempts in a single stifle at one time point (Dog 1 at T0). PGE_2_ concentrations varied significantly based on number of aspiration attempts (Table 2).

**Table 2:** Effect of number of aspiration attempts on synovial fluid PGE_2_ concentration

Aspiration Attempts	Sample size	Median PGE_2_ (pg/mL)	Range
1	62	304.3^a^	58.3–1722.4
2	7	244.5^a^	110.9–2425.9
3	5	734.9^b^	306.7–5032.2

Values with different superscript letters are significantly different

^a^One attempt was not significantly different than two attempts (p = 0.984)

^b^Two attempts were significantly different than three attempts (p = 0.041)

^b^One attempt was significantly different than three attempts (p = 0.005)

Based on the analysis of number of attempts, samples requiring ≥ 3 aspiration attempts were omitted and the data re-analyzed (Table 3). In total, 11/80 samples were omitted. Following re-analysis, PGE_2_ concentrations in the bupivacaine group increased significantly over time (p = 0.008). In the bupivacaine group, PGE_2_ concentrations in samples collected at T24 (p = 0.003) and T48 (p = 0.041) were significantly higher compared to T0. There were no changes in PGE_2_ concentrations in saline-treated joints over time (p = 0.207). There were no significant differences between saline- and bupivacaine-injected joints at any time point (p = 0.261–0.949).

**Table 3:** PGE_2_ concentrations in canine stifle synovial fluid following bupivacaine or saline injection in stifles requiring ≤ 2 attempts

Time	Treatment	Sample size	Median (pg/mL)	Range
T0	Bupivacaine*	5	110.9^#,§^	58.3–382.1
T0	Saline	7	184.8	96.2–434.8
T30	Bupivacaine	6	239.1	128.9–338.3
T30	Saline	8	270.0	91.7–430.1
T60	Bupivacaine	6	362.4	142.3–669.1
T60	Saline	8	468.3	172.3–1074.2
T24	Bupivacaine	8	726.2^#^	197.8–1675.8
T24	Saline	8	446.4	159.8–910.3
T48	Bupivacaine	6	391.6^§^	210.0–1722.4
T48	Saline	7	274.3	118.5–2425.9

* Significant difference over time within the bupivacaine group (p = 0.008)

^#^ T24 is significantly different from T0 (p = 0.003)

^§^ T48 is significantly different from T0 (p = 0.041)

Finally, a post hoc analysis was performed to evaluate the effect of ≥ 3 attempts on all *subsequent* synovial fluid samples. Using a mixed model ANOVA, all samples collected before ≥ 3 attempt-samples were compared to samples collected after ≥ 3 attempt-samples, excluding samples that required ≥ 3 attempts. No evidence was found to suggest that ≥ 3 aspiration attempts influenced the PGE_2_ concentration in subsequent samples, regardless of whether all stifles were analyzed together (p = 0.221) or whether bupivacaine and saline stifles were analyzed separately (p = 0.092 and 0.582, respectively).

### Discussion

In normal canine stifles in our study, a single intra-articular injection of bupivacaine did not result in increased synovial fluid PGE_2_ concentrations compared to saline-injected controls regardless of number of aspiration attempts. Following re-analysis, PGE_2_ concentrations in the bupivacaine group increased significantly over time (p = 0.008). Measurement of synovial fluid PGE_2_ concentrations was complicated by the predictable increase in synovial fluid volume following injection, as well as the variable number of aspiration attempts for each joint. Our data indicate that synovial fluid PGE_2_ concentration is highly sensitive to number of aspiration attempts. This is an important consideration when performing arthrocentesis in clinical cases and in designing future studies.

There is conflicting evidence regarding the expected inflammatory or anti-inflammatory effects of local anesthetics on the synovium. In an in vitro canine co-culture model of the synovial joint, 0.5% bupivacaine inhibited an expected rise in PGE_2_ following addition of IL-1β to the culture medium [8]. Neurogenic inflammation was reduced following a 30-min continuous infusion (200 μL/min) of low concentrations of either lidocaine (0.02%) or bupivacaine (0.05%) in inflamed rat stifles [9]. These two studies suggest an anti-inflammatory effect of local anesthetics. In contrast, other studies have documented evidence of synovitis after intra-articular local anesthetic injection [10–12]. Similarly, when corrected for number of aspiration attempts, our results identified a significant increase in PGE_2_ from baseline within the bupivacaine group, suggesting an inflammatory response following injection. Confidence in this conclusion is weak considering the lack of significance between treatment and control groups.

An unexpected observation in our study was the significant increase in synovial fluid PGE_2_ concentrations in samples that required repeated arthrocentesis attempts (≥ 3 attempts in a single time point). Frequent needle penetration of the synovium is the most likely source of this inflammation rather than the treatment agent, because it occurred in both treatment groups. Increased PGE_2_ concentrations in synovial fluid following arthrocentesis is also documented in horses and as a result, those authors suggest separating repeated aspirations by 7 days to account for the inflammation created by arthrocentesis [13]. In our study, the small size of the joint and resulting small synovial fluid volume available for sampling likely contributed to the necessity for repeated arthrocentesis.

We anticipated that the normal Beagle stifle would contain a small synovial fluid volume (1 mL fluid/12.5 kg dog). As a result, following aspiration at T0 and the subsequent infusion of the treatment volume (~ 2.5 mL), later PGE_2_ concentrations would be decreased due to dilution by the larger relative fluid volume. In a previous study evaluating how quickly bupivacaine elutes from the stifle following intra-articular administration, a similar dilutional effect was noted following treatment injection, and our pre-injection fluid volume approximation was calculated based on those methods [7]. Therefore, 0.08 mL/kg synovial fluid volume was approximated for each stifle and defined as Vi [7].

The effect of aspiration attempts influencing our data resulted in 11/80 samples being excluded from the final analysis. Dogs 1 and 5 were particularly difficult, contributing to 8/11 omitted samples. The difficulty associated with Dog 1 was attributed to the learning curve associated with the procedure while Dog 5 was attributed to his small size and resultant small initial synovial fluid volume.

A time- and dose-dependent effect of bupivacaine on chondrotoxicity in vitro is reported [1, 4, 14–16]; however, to our knowledge, chondrolysis has not been reported in the veterinary literature following a single intra-articular dose of bupivacaine in vivo. A pharmacologic study of a single intra-articular dose of bupivacaine identified a rapid decrease in bupivacaine concentrations (11–25% of baseline bupivacaine concentration) 30 min after injection in dogs [7]. Given the rapid clearance of a single intra-articular dose of bupivacaine and the results of our study, a single injection of bupivacaine is unlikely to cause sufficient synovitis to be the sole cause of chondrolysis. Our study establishes that repeated arthrocentesis results in elevated synovial fluid PGE_2_ concentrations.

## Limitations

An important limitation is the use of normal stifles; the results of our study may not accurately portray the response within a diseased joint (i.e. osteoarthritic joint), when bupivacaine is most likely to be used therapeutically. Elevated PGE_2_ concentrations and synovitis are a common feature of osteoarthritis and may either blunt the joint’s response to bupivacaine or prime the joint for an enhanced response to bupivacaine injection [17, 18]. A second limitation was the small available synovial fluid volume at T0 in Beagles with normal stifles. This presented a particular challenge for aspiration, resulting in additional aspiration attempts that affected our data, as described.

## Abbreviations

PGE_2_: prostaglandin E_2_
ELISA: enzyme-linked immunosorbent assay
ANOVA: analysis of variance

## Footnotes

1 Excel 2016, Microsoft Corporation, Redmond, WA, USA.

2 Marcaine 0.5% (preservative free), Hospira, Lake Forest, IL, USA.

3 Cayman Chemical Company, Ann Arbor, MI, USA.

4 Spectramax 5, Molecular Device, Sunnyvale, CA, USA.

5 SAS version 9.4, SAS Institute Inc., Cary, NC, USA.
